# Supplementary material for: Loss of miR-210 leads to progressive retinal degeneration in Drosophila melanogaster
Source: Life Sci Alliance. 2019 Jan 22;2(1):e201800149. doi: 10.26508/lsa.201800149 (PMC6343102; doi:10.26508/lsa.201800149)
Supplement: Supplementary file 1 [file LSA-2018-00149_Table_S1.docx]

**Supplemental Table 1 Cloning strategy for vectors generated in this study including primers used.** Underlined bases highlight the used restriction site. **Fat** bases highlight siRNA or guideRNA sequences within the primers. small bases indicate mutations.

| Construct | Vector backbone | Primer | Primer sequence | Strategy |
| --- | --- | --- | --- | --- |
| pCFD4 miR-210 | pCFD4 | CW025 | TATATAGGAAAGATATCCGGGTGAACTTC**GCCAATGATGATTAGTGTGCC**GTTTTAGAGCTAGAAATAGCAAG | pCFD4 Cloning  Port et al. (2014) |
|  |  | CW026 | ATTTTAACTTGCTATTTCTAGCTCTAAAAC**TAGAGATTGCCGCCTAATGC**GACGTTAAATTGAAAATAGGTC |  |
| pBS miR-210∆ | pBluescript SK+ | CW032 | ATCTCGAGAACCAAAATTCGTTGATCCTTTCCTGAAAT | 1) Amplification of up-stream region by CW032 + 033. Restriction digest by XhoI and EcoRI  2) Amplification of down-stream region by CW034 + 035. Restriction digest by SpeI + EcoRI  3) Mutations in the PAM sites by QuikChange Mutagenesis II Kit (CW036 + 037) |
|  |  | CW033 | ATGAATTCCGGTTCCGGACGTGGTCGGT |  |
|  |  | CW034 | ATGAATTCAGCCATTGTTCCATCATCGTGGT |  |
|  |  | CW035 | ATACTAGTATCCTTAAGTTTCAGATCTAGTGACGAATGC |  |
|  |  | CW036 | CAATGATGATTAGTGTGCCAGtCGACCGACCAGCAATGG |  |
|  |  | CW037 | CAGATTGCCGCAGAGAAGaCGTAGAGATTGCCGCCTAATG |  |
| pBS miR-210∆ GFP | pBS miR-210∆ | CW041 | ATGAATTCCAAAATGGTGAGCAAGGGCGAGGAGCTGTTCAC | 1) Amplification of eGFP with CW041 + 042  2) Restriction digest by EcoRI |
|  |  | CW042 | ATGAATTCCTACTTGTACAGCTCGTCCATGCGGAGAGTGAT |  |
| pCFD3-miR-210 | pCFD3 | CW028 | GTCGACTC**TTGTGCGTGTGACAG** | pCFD3 Cloning  Port et al. (2014) |
|  |  | CW029 | AAAC**CTGTCACACGCACAA**GAGT |  |
| pUAST attb Dgk | pUAST attb | CW382 | ATACTAGTATGAATATTGGCATCGCAGCACCGAAA | Restriction digest by SpeI and HindIII |
| pMIR Fasn1 | pMIR Report | Gene Synthesis | GCTAGAAAGAGGATTAGAATAAGAGTTGTGAATGTGGCATAGGCCCACTACCCACACGATCATCACCCTCTCACCGTCACTCACCCAATACTCGGGTTCTATGCACCATGTGGAACCACGGAACACATCGACACCTAGTTAGCATACTTAGTGAATGAATTTACGTTGTTGAGCTGCAAGGAATGGCTCGCCCCATCCCAGTTACGCACAGCTAGAAGAGATAAGTTAACAGATTGGATTTGCGTCGATCGGATTGAATCTGCCTAACTGCTGTCTTGCAGGGCTGTAGGCTTCGGTCCAAAATTAAGTCTACAGTCTCTGCGATTCCGGGCAGTTGAAATGATGCAAGATGAGAGGCTCAGTCCATAACACTTAACGCTTTGTGCACCGTACAATTTAGTACCCGACATTCCTTGAGAGCTAGACGGCACCAGACGTCCCAACTTACCAAATATATCTTTTATGCTCTATCTATAATGTATGTCGCATATTCTAGTTTT | Restriction digest by SpeI and Hind III |
| pMIR Fasn1 mutated | pMIR Report | Gene Synthesis | GCTAGAAAGAGGATTAGAATAAGAGTTGTGAATGTGGCATAGGCCCACTACCCACACGATCATCACCCTCTCACCGTCACTCACCCAATACTCGGGTTCTATGCACCATGTGGAACCACGGAACACATCGACACCTAGTTAGCATACTTAGTGAATGAATTTACGTTGTTGAGCTGCAAGGAATGGCTCGCCCCATCCCAGTTaaccggtGCTAGAAGAGATAAGTTAACAGATTGGATTTGCGTCGATCGGATTGAATCTGCCTAACTGCTGTCTTGCAGGGCTGTAGGCTTCGGTCCAAAATTAAGTCTACAGTCTCTGCGATTCCGGGCAGTTGAAATGATGCAAGATGAGAGGCTCAGTCCATAACACTTAACGCTTTGTGCACCGTACAATTTAGTACCCGACATTCCTTGAGAGCTAGACGGCACCAGACGTCCCAACTTACCAAATATATCTTTTATGCTCTATCTATAATGTATGTCGCATATTCTAGTTTT | Restriction digest by SpeI and Hind III |
