# Supplementary material for: Loss of miR-210 leads to progressive retinal degeneration in Drosophila melanogaster
Source: Life Sci Alliance. 2019 Jan 22;2(1):e201800149. doi: 10.26508/lsa.201800149 (PMC6343102; doi:10.26508/lsa.201800149)
Supplement: Supplementary file 2 [file LSA-2018-00149_Table_S2.docx]

**Supplemental Table 2: q-RT-PCR primers used in this study (SYBR Green and TaqMan)**

| **Target gene** | **Forward primer** | **Sequence (for)** | **Reverse primer** | **Sequence (rev)** | **TaqMan Probe** |
| --- | --- | --- | --- | --- | --- |
| Rpl32 | SOL268 | ATATGCTAAGCTGTCGCACAAATGG | SOL269 | GATCCGTAACCGATGTTGGGCA |  |
| Dgk | CW343 | TGCACTCCTTCTTCACCGAC | CW345 | TGCACTCCTTCTTCACCGAC |  |
| miR-210 |  |  |  |  | 005997_mat |
| Scylla |  |  |  |  | Dm01798373_s1 |
| snoR442 |  |  |  |  | 001742 |
